# Immortalized human choroid plexus endothelial cells enable an advanced endothelial-epithelial two-cell type *in vitro* model of the choroid plexus

**DOI:** 10.1016/j.isci.2022.104383

**Published:** 2022-05-10

**Authors:** Walter Muranyi, Christian Schwerk, Rosanna Herold, Carolin Stump-Guthier, Marko Lampe, Petra Fallier-Becker, Christel Weiß, Carsten Sticht, Hiroshi Ishikawa, Horst Schroten

**Affiliations:** 1 Pediatric Infectious Diseases, Department of Pediatrics, Medical Faculty Mannheim, Heidelberg University, Mannheim, Germany; 2 European Center for Angioscience, Medical Faculty Mannheim, Heidelberg University, Mannheim, Germany; 3 Advanced Light Microscopy Facility, European Molecular Biology Laboratory, Heidelberg, Germany; 4 Institute of Pathology and Neuropathology, University of Tübingen, Tübingen, Germany; 5 Department of Medical Statistics and Biomathematics, University Medical Center Mannheim, Heidelberg University, Heidelberg, Germany; 6 Core Facility Next Generation Sequencing, Medical Faculty Mannheim, Heidelberg University, Mannheim, Germany; 7 Laboratory of Clinical Regenerative Medicine, Department of Neurosurgery, Faculty of Medicine, University of Tsukuba, Tsukuba, Japan

**Keywords:** Biological sciences, Cell biology, Biological sciences research methodologies, Biology experimental methods

## Abstract

The choroid plexus (CP) is a highly vascularized structure containing endothelial and epithelial cells located in the ventricular system of the central nervous system (CNS). The role of the fenestrated CP endothelium is under-researched and requires the generation of an immortalized CP endothelial cell line with preserved features. Transduction of primary human CP endothelial cells (HCPEnC) with the human telomerase reverse transcriptase (hTERT) resulted in immortalized HCPEnC (iHCPEnC), which grew as monolayer with contact inhibition, formed capillary-like tubes in Matrigel, and showed no colony growth in soft agar. iHCPEnC expressed pan-endothelial markers and presented characteristic plasmalemma vesicle-associated protein-containing structures. Cultivation of iHCPEnC and human epithelial CP papilloma (HIBCPP) cells on opposite sides of cell culture filter inserts generated an *in vitro* model with a consistently enhanced barrier function specifically by iHCPEnC. Overall, iHCPEnC present a tool that will contribute to the understanding of CP organ functions, especially endothelial-epithelial interplay.

## Highlights

• Generation of an immortalized human choroid plexus endothelial cell line (iHCPEnC)
• iHCPEnC immortalized by telomerase maintain essential endothelial properties
• The mRNA expression profile distinguishes iHCPEnC from other endothelial cell types
• iHCPEnC enhance the barrier function of a choroid plexus epithelium in coculture

## Graphical abstract

**Figure:**
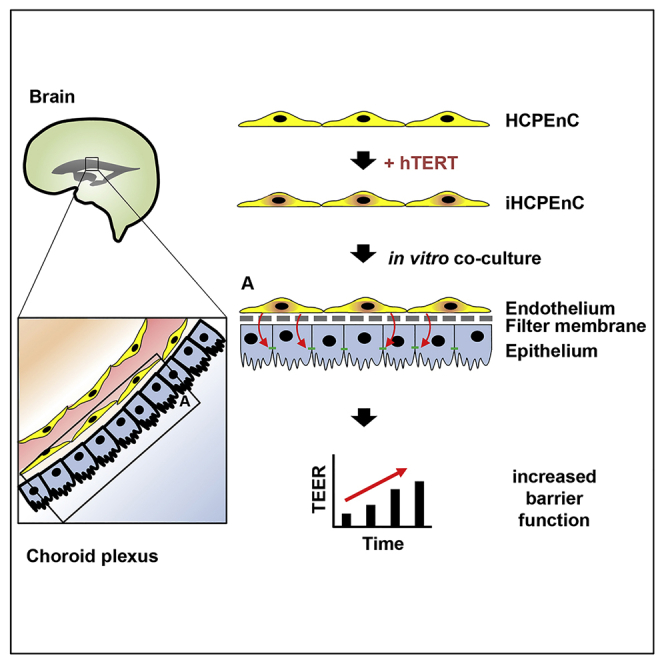

## Introduction

The human choroid plexus (CP) is a highly vascularized structure containing endothelial, epithelial, immune, mesenchymal, glial, and neuronal cells located in the ventricles of the brain. It plays important roles in cerebrospinal fluid (CSF) production, brain development, aging, neuroimmune interactions, the pathogenesis of CNS infections, neurodegenerative diseases, and the constitution of a blood-CSF barrier (BCSFB) (Wolburg and Paulus, 2010; Kaur et al., 2016; Perez-Gracia et al., 2009; Wilson et al., 2010; Serot et al., 2001). In contrast to the tight epithelium, which provides the BCSFB function of the CP, the capillaries of the CP are constituted by fenestrated endothelial cells that separate the lumen from the stroma.

The understanding of CP functions were established with the help of *in vitro* models based on primary cells isolated from different species or mostly animal cell lines of tumor origin (Strazielle and Ghersi-Egea, 2011; Tenenbaum et al., 2013; Schwerk et al., 2015). Noteworthy, many of the functions of the CP are related to the ability of this organ to generate a barrier between the blood and the CSF. Beside animal models, functional CP *in vitro* cultures, based on the barrier properties and restricted paracellular permeability, have been established with CP epithelial cells that are cultured on microporous inserts. These include human epithelial CP papilloma (HIBCPP) cells that at confluence develop polarity and high (500–800 Ω cm^2^) transepithelial electrical resistance (TEER), making them useful for studying therapeutic drug transport as well as pathogen and immune cell passage (Strazielle and Ghersi-Egea, 2011; Tenenbaum et al., 2013; Dinner et al., 2016).

It is assumed that an important role in maintaining organ functions is played by organ specific endothelial cells (Augustin and Koh, 2017), arguing for a potential significance of the CP endothelium at the BCSFB. Although the HIBCPP cells present a functional human cell line of the CP epithelium (Ishiwata et al., 2008; Schwerk et al., 2012), a cell line of human CP endothelial cells (HCPEnC) is still missing with the consequence of highly underrepresented research on the CP endothelium. In addition, coculture systems employing both epithelial and endothelial cells of the CP are required. However, the limited amount of cellular passages of primary cells and the inevitable variability between different primary cell cultures led to the necessity to generate a cell line presenting characteristic endothelial markers as well as reproducible morphology and gene expression. The endothelial cells of the CP are fenestrated and the tight junctional proteins Claudin 1 (CLDN1), Claudin 5 (CLDN5), Occludin (OCLN), and Zonula occludens 1 (ZO1) are detected in rats (Lippoldt et al., 2000). The proteinaceous substrate of endothelial fenestration is the plasmalemma vesicle-associated protein (PLVAP) that assembles into stomatal and fenestral diaphragms covering caveolae, transendothelial channels (TEC), and fenestrae (Stan et al., 1997, 1999) and is expressed in the CP endothelium of mice (Dani et al., 2021). The main function of PLVAP is associated with regulation of cell layer permeability and transendothelial extravasation of immune cells (Keuschnigg et al., 2009; Bosma et al., 2018).

The limited cell division of primary human endothelial cells can be overcome by ectopic expression of human telomerase reverse transcriptase (hTERT), which induces immortalization of cells (Harley et al., 1990; Bodnar et al., 1998). The ectopic expression of the catalytic domain of hTERT, alone or in combination with a viral oncogene (simian virus 40 (SV40) large T antigen), was efficient in immortalization of human fibroblasts, retinal pigment epithelial cells, and brain microvascular endothelial cells (Jiang et al., 1999; Weksler et al., 2005). The immortalized microvascular endothelial cell lines express typical markers as the platelet endothelial cell adhesion molecule (PECAM1), vascular endothelial cadherin (CDH5), and von Willebrand factor (VWF) (Weksler et al., 2005).

We describe here the generation and characterization of immortalized HCPEnC (iHCPEnC) by ectopic expression of hTERT in primary cells using a lentiviral vector system without additional support by a viral oncogene. RNA sequencing (RNA-seq) analysis revealed that iHCPEnC retained an RNA expression profile very similar to the primary HCPEnC and different from that of other endothelial cell types as Human Brain Microvascular Endothelial Cells (HBMEC) and Human Umbilical Vein Endothelial Cells (HUVEC). iHCPEnC express the endothelial markers PECAM1 and VWF and retained important PLVAP-based morphological characteristics of primary CP endothelial cells, such as caveolae and fenestrae. The generation of iHCPEnC offers a reproducible *in vitro* model of the CP endothelium susceptible to genetic manipulation. By establishing an advanced coculture model consisting of CP endothelial (iHCPEnC) and epithelial (HIBCPP) cells grown on opposite sides of filter inserts, we generated an invaluable tool that will help to dissect the endothelial-epithelial interplay in the CP.

## Results

### Generation of a non-transformed CP endothelial cell line

**Figure 1:**
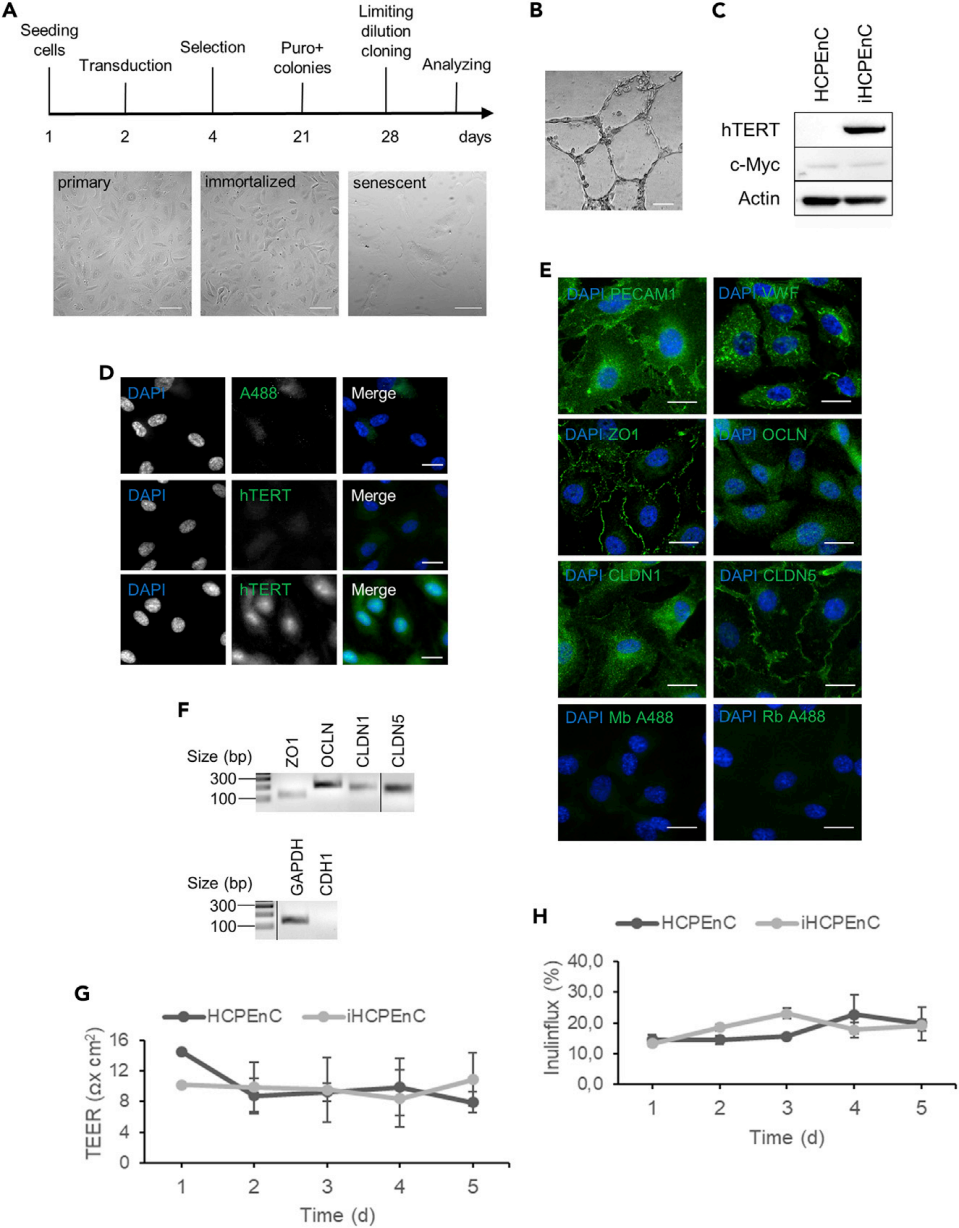
Morphological and functional characteristics of the iHCPEnC (A) Experimental workflow for the generation of iHCPEnC and phase contrast image of primary HCPEnC, hTERT-expressing iHCPEnC, and primary senescent HCPEnC. The immortalized cell line grew as a monolayer with characteristic endothelial cellular morphology comparable to primary HCPEnC. Senescent primary HCPEnC revealed an enlarged and flattened morphology. Scale bar, 100 μm. (B) Phase contrast microscopy image of the capillary-like network of iHCPEnCs maintained 72 h on Matrigel. Scale bar, 100 μm. (C) Immunoblot analysis of primary and iHCPEnCs cells detecting expression of hTERT and endogenous c-Myc. (D) Detection of hTERT by indirect immunofluorescence. Primary HCPEnC and iHCPEnC grown on coverslips were stained for detection of hTERT. Only iHCPEnC showed a peri- and nuclear expression of the catalytic unit of hTERT. Cell nuclei were visualized using DAPI. As negative control, immunofluorescence without primary antibody was performed. Scale bar, 20 μm. (E) Immunofluorescence of pan-endothelial markers and tight junction proteins. iHCPEnC grown on coverslips were stained for detection of PECAM1 and VWF as endothelial markers and ZO1, OCLN, CLDN1, and CLDN5 as markers for tight junction proteins as indicated (green). Nuclei were visualized using DAPI (blue). As negative control, immunofluorescence without primary antibody was performed. Scale bar, 20 μm. (F) Agarose gel electrophoresis of RT-PCR products of tight junction protein transcripts. The indicated transcripts were detected in two independent reactions; a typical result is shown. Epithelial cadherin (CDH1), which is not expressed in endothelial cells, was used as negative control, GAPDH served as positive control. (G) TEER values of iHCPEnC seeded on filter inserts were monitored over a period of 5 days after confluence. Data are represented as mean ± SEM of three independent experiments performed in triplicates. (H) FITC-inulin flux across iHCPEnC seeded on filter inserts and grown to confluence was measured during a time-course of 5 days. The FITC-Inulin tracer solution was added to the upper compartment of the filter for 4 h. Data shown are represented as mean ± SEM of two independent experiments each performed in triplicates.

Primary human CP endothelial cells were derived from a single donor of the age of 18. The cells were immortalized by lentiviral transduction of hTERT and the obtained immortalized HCPEnC (iHCPEnC) revealed endothelial morphology after 60 population doubling time (30 passages), showed no senescence characterized by enlarged, flattened cells, and grew strictly as monolayer (Figure 1A). iHCPEnC failed to form colonies in soft agar indicating a nononcogenic transformation and they retained the capacity of angiogenesis on Matrigel (Figure 1B). hTERT expression of the iHCPEnC was examined by immunoblot, detecting a protein of ∼127 kDa in size (Figure 1C), and by immunofluorescence detecting a nuclear staining (Figure 1D). Ectopic expression of hTERT can lead to activation of the c-Myc oncogene in some cells, thereby sustaining cell proliferation independently from the telomerase expression (Wang et al., 2000). We examined the expression of c-Myc in iHCPEnC and compared it with that in the primary HPCEC by immunoblotting, and no elevated c-Myc expression was observed in iHCPEnC (Figure 1C).

### iHCPEnC expresses endothelial markers and tight junction proteins

iHCPEnC was analyzed for the expression and cellular distribution of pan-endothelial markers (PECAM1 and VWF) and tight junction proteins by indirect immunofluorescence. In endothelial cells, PECAM1 displays a cellular localization at the plasma membrane, and VWF is found as cytoplasmic granules, respectively (Lenting et al., 2015). iHCPEnC were found positive for both markers with typical cellular distribution (Figure 1E). Several tight junction proteins, such as ZO1 (submembranous) as well as OCLN, CLDN1, and CLDN5 (transmembranous), are expressed in the CP of rats. A weaker CLDN1 expression and a prominent immunohistological signal of CLDN5 was described (Lippoldt et al., 2000). OCLN was not consistently detected at the cell-cell contact sites in contrast to CLDN1, CLDN5, and ZO1, which were continuously observed at cell junctions (Figure 1E).

The expression of tight junction proteins in iHCPEnC was analyzed by qualitative RT-PCR from total RNA. As negative control, epithelial cadherin (CDH1) was used. Transcripts of ZO1, OCLN, CLDN1, and CLDN5 were detected. Consistently, a stronger CLDN5 signal in comparison to CLDN1 was obtained (Figure 1F).

### iHCPEnC displays no considerable barrier function

To determine whether the endothelial cells might also contribute to the TEER at the BCSFB, iHCPEnC were seeded on filter inserts coated with Attachment Factor^TM^ and grown in endothelial medium. TEER measurements of primary HCPEnC and iHCPEnC were performed over 5 days following confluence, verified by microscopic inspection, and the values determined were between 10 and 20 Ω × cm^2^, indicating that iHCPEnC did not develop a considerable TEER within the investigated time frame (Figure 1G).

Barrier function is also characterized by a restricted paracellular permeability of the cell layer. To measure the paracellular permeability of primary HCPEnC and iHCPEnC, cells were seeded on filter inserts and after confluence FITC-labeled Inulin was added as macromolecular tracer (average molecular weight of 3000–6000) to the upper compartment for 4 h. Evaluation of fluorescence was performed over 5 days and started after confluence of the cells and yielded a paracellular flux from around 15%–25%, again indicating no considerable barrier function (Figure 1H).

### The transcriptome of iHCPEnC is similar to primary HCPEnC and different from other endothelial cell lines

**Figure 2:**
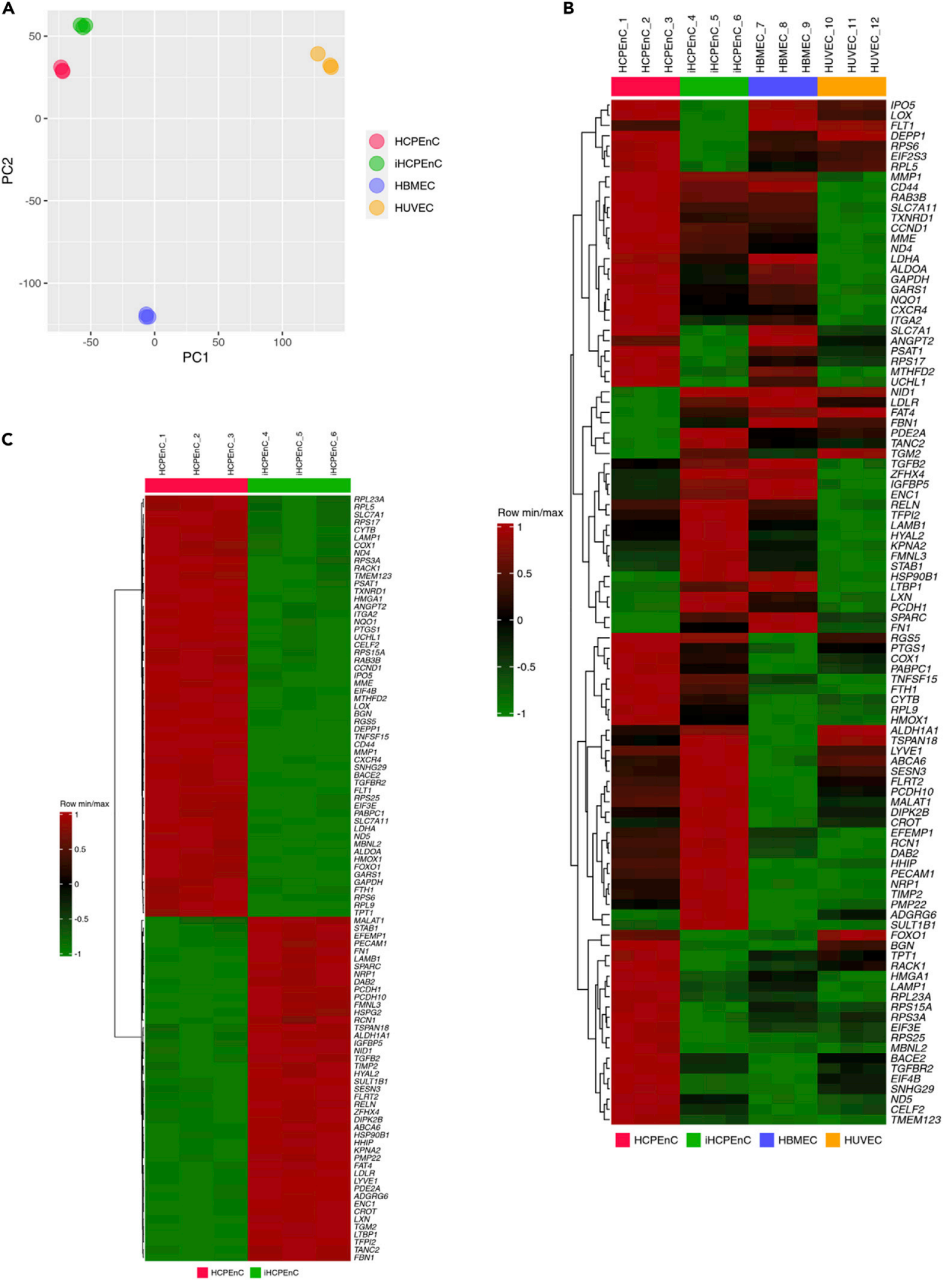
Transcriptome similarity between iHCPEnC and primary HCPEnC, HBMEC and HUVEC, respectively (A) A principal component analysis (PCA) of all four cell lines based on the 18,025 genes measured with RNA-seq. Principal component (PC) 1 splits HUVEC from the other three cell lines, whereas PC2 splits HBMEC from the other three cell lines. A close similarity is demonstrated between HCPEnC and iHCPEnC compared to HBMEC or HUVEC. (B) Heatmap generated from the top 100 most significant genes of all comparisons HCPEnC, iHCPEnC, HBMEC, and HUVEC. (C) Heatmap generated from the top 100 most significant genes between HCPEnC and iHCPEnC.

To investigate to which extent the iHCPEnC resemble the primary HCPEnC, we performed an RNA-seq analysis using total RNA isolated from primary cells and iHCPEnC. Furthermore, to determine whether iHCPEnC display a characteristic expression pattern, the transcriptome of two different endothelial cell types, HBMEC (a blood-brain barrier-derived cell line) and HUVEC (derived from the endothelium of veins from the umbilical cord), was analyzed for comparison. A principal component analysis (PCA) was performed to demonstrate the close similarity between the primary and immortalized CP endothelial cells compared to HBMEC or HUVEC (Figure 2A). This result is confirmed by the heatmaps shown in Figures 2B and 2C. Although a heatmap based on the top 100 significant genes between all four cell types shows the similarity between HCPEnC and iHCPEnC and a clear difference from HBMEC and HUVEC (Figure 2B), the similarity between HCPEnC and iHCPEnC is further confirmed by a heatmap based on the top 100 significant genes between these two cell types (Figure 2C).

To obtain information concerning differences in the biology of the four endothelial cell lines, Gene Set Enrichment Analyses (GSEA) based on the Gene Ontology Biological Processes method (GOBP) were performed based on t-values obtained with student’s *t*-tests between the cell lines. The GSEA comparing primary HCPEnC with iHCPEnC (see Table S1, related to Figure 2C and STAR Methods) resulted in the identification of 805 gene sets. In contrast, GSEA comparing iHCPEnC with HBMEC (see Table S2, related to Figure 2B and STAR Methods) or HUVEC (see Table S3, related to Figure 2B and STAR Methods) identified 820 and 870 gene sets, respectively.

**Figure 3:**
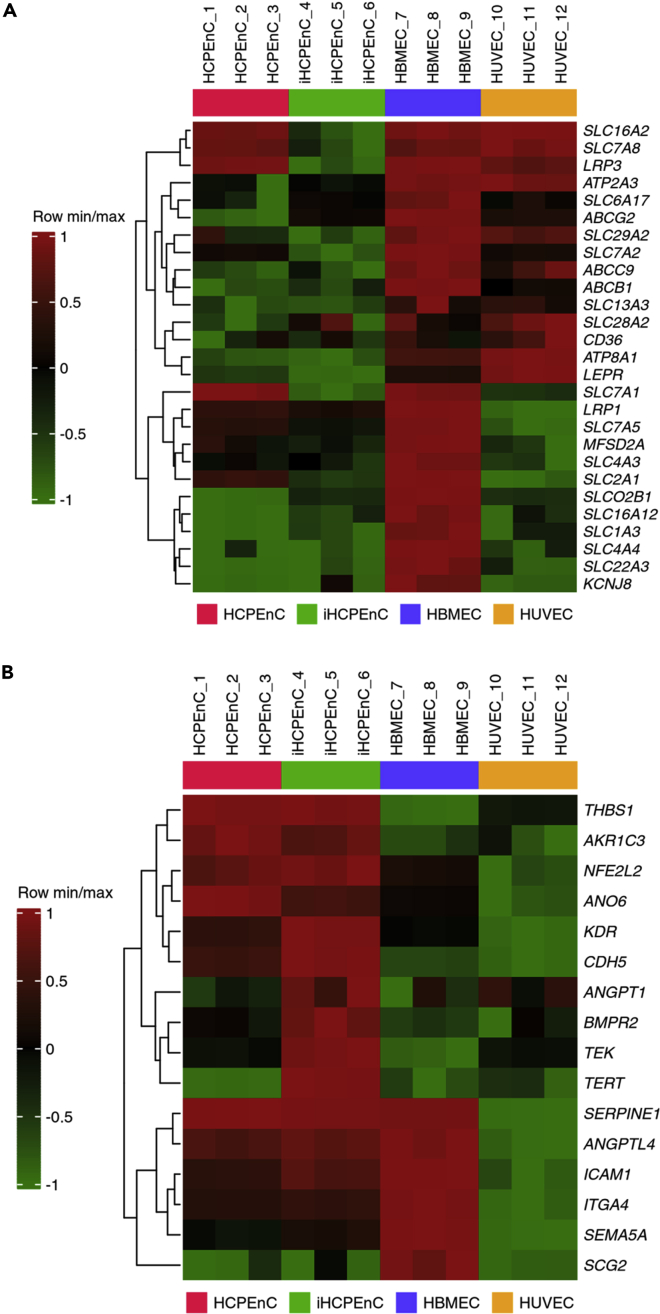
Transcriptome similarity between iHCPEnC and primary HCPEnC, HBMEC, and HUVEC, respectively (A) Heatmap generated with genes selected from the gene set “transport across blood-brain barrier”. (B) Heatmap generated with genes selected from the gene set “endothelial cell apoptotic process”.

Following the GSEA, genes from gene sets significantly different between iHCPEnC and HBMEC or iHCPEnC and HUVEC, respectively, were selected for generation of heat maps. Figure 3A shows a heatmap generated with genes selected from the gene set “transport across blood-brain barrier,” which displays a consistently higher expression in HBMEC compared to iHCPEnC. Figure 3B shows a heatmap generated with genes selected from the gene set “endothelial cell apoptotic process,” which displays a consistently lower expression in HUVEC compared to iHCPEnC.

### iHCPEnC exhibits PLVAP-associated cellular structures

Single cell RNA sequencing, combined with spatial RNA and protein mapping of the endothelial cells of the mouse CP, revealed a repertoire of cellular markers essential for development and physiology of the CP (Dani et al., 2021). Among these markers was PLVAP, the only protein known to be involved in the formation of fenestral and stomatal diaphragms. Expression of PLVAP predicts the presence of PLVAP-associated cellular structures as caveolae, TEC, and fenestrae (Stan et al., 1997, 1999; Herrnberger et al., 2014). *PLVAP* is expressed in endothelia mainly in cells which fenestrate and are involved in regulation of cellular permeability and extravasation of immune cells (Keuschnigg et al., 2009).

Besides *PLVAP*, we selected additional genes, which were subjected to qualitative RT-PCR in iHCPEnC. *ESM1* (Endothelial Cell-Specific Molecule 1) encodes for an endothelial cell protein involved in signaling, adhesion, and migration processes, and was found coexpressed with PLVAP in mouse CP endothelial cells (Dani et al., 2021). *MFSD2A* (Major Facilitator Superfamily Domain Containing 2A) encodes for a sodium-dependent lysophosphatidylcholine transporter protein important for brain development and function (Dani et al., 2021). The product of *LYVE1*, the Lymphatic Vessel Endothelial Hyaluronan Receptor 1, binds hyaluronan and was shown to associate with PLVAP in liver sinusoidal endothelial cells (Auvinen et al., 2019). The *ACTA2* gene product (Actin Alpha 2) is involved in vascular contractility and was used as marker in murine CP explants to differentiate in combination with VWF between Acta2^+^ (arterial) and ACTA2^-^ (venous) blood vessels (Dani et al., 2021). The *VEGFR* (Vascular Endothelial Growth Factor Receptor) *1-3* gene products represent receptors regulating PLVAP protein expression and intracellular distribution. VEGFR1 binds VEGF-A and VEGF-B and plays an important role in angiogenesis, VEGFR2 binds VEGF and is important for endothelial proliferation, migration, and formation of tubular structures, and VEGFR3—a tyrosine kinase receptor for VEGF C and D—is involved in endothelial cell proliferation, migration, and regulation of angiogenic sprouting. *GAPDH* (Glyceraldehyde-3-Phosphate Dehydrogenase) was used as control.

**Figure 4:**
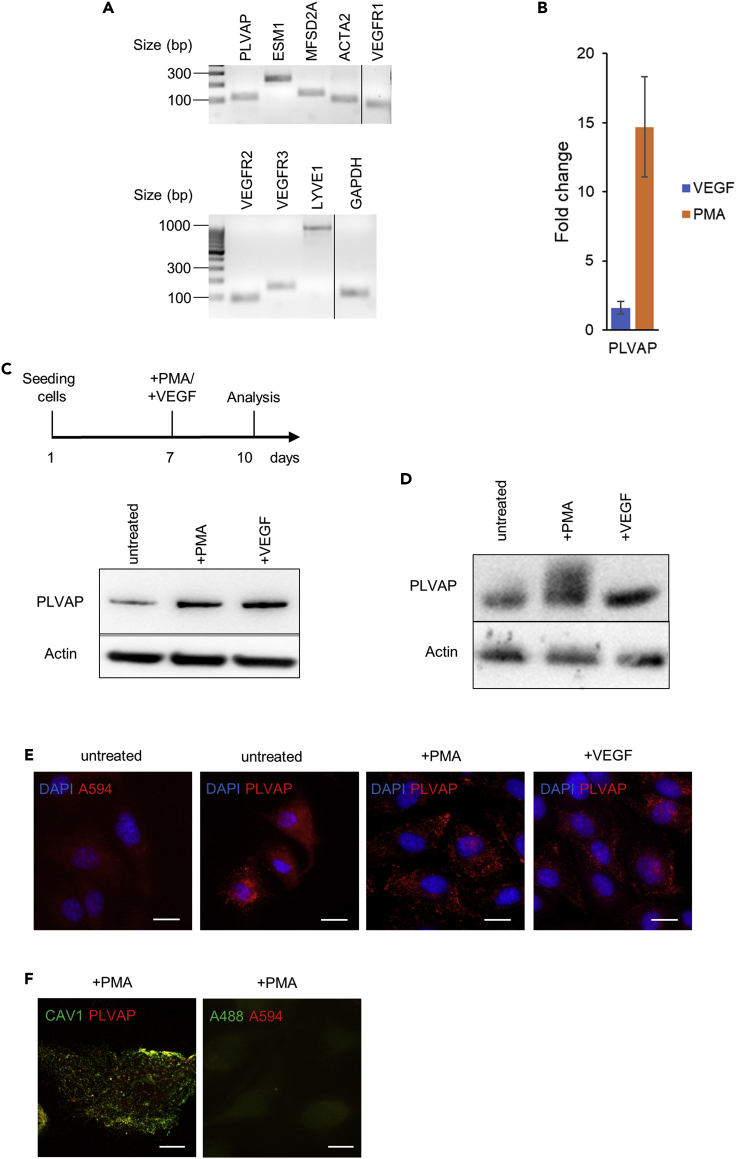
The iHCPEnC express the PLVAP protein (A) RT-PCR detection of representative BCSFB transcript expression in iHCPEnC after treatment with PMA for 3 days iHCPEnC were seeded on a 6 well plate and grown to confluence. Total RNA was extracted and subjected to quantitative RT-PCR. Transcript expression was analyzed by gel electrophoresis of RT-PCR products. (B) QPCR quantification of increased PLVAP expression evaluated by the ΔΔCT method. For the experiment the following numbers of data were used: PMA, n = 2; VEGF, n = 3. Data are represented as mean ± SEM. (C) Experimental workflow for the generation of samples and (D) Detection of PLVAP protein expression in iHCPEnC by Western blotting. Total protein lysates of untreated iHCPEnC or iHCPEnC treated with PMA (50 nM) or VEGF (40 ng/mL) for 3 days were analyzed by Western blotting with a mouse monoclonal (C) and rabbit polyclonal (D) antibody against PLVAP, respectively. (E) Indirect immunofluorescence of PLVAP expression in iHCPEnC. iHCPEnC seeded on coverslips and left either untreated or were treated with PMA (50 nM) or VEGF (40 ng/mL) for 3 days, protein was detected by indirect immunofluorescence using an antibody against PLVAP. Immunofluorescence without primary antibody was performed and used as negative control. Scale bar, 20 μm. (F) Colocalization of PLVAP with CAV1 by indirect immunofluorescence. iHCPEnC were seeded on glass coverslips and treated with PMA for 3 days. Fixed cells were permeabilized and stained with anti-PLVAP (red) and anti-CAV1 (green). As negative control, immunofluorescence without primary antibodies was performed. Scale bar, 10 μm.

Endothelial fenestrae and caveolar or stomatal diaphragms are inducible plasma membrane structures that can be induced in cell culture by PMA or VEGF. It was shown that endothelial cells, which demonstrate fenestration when tissues from mice are analyzed, reduce these surface structures under cell culture conditions, an effect that can be reversed by addition of PMA (Milici et al., 1985; Stan et al., 2004). PLVAP is upregulated when cells are stimulated with 50 nM phorbol-12-myristate-13-acetate (PMA) or are exposed to VEGF (40 ng/mL) (Stan et al., 2004; Hamilton et al., 2019). Total RNA from iHCPEnC were treated with PMA to achieve similar expression conditions for potential candidate interacting proteins for 3 days and were subjected to RT-PCR with specific primers representative for CP-derived endothelial cells (Dani et al., 2021; Hamilton et al., 2019). Expression of all tested transcripts was detected in iHCPEnC (Figure 4A).

Employing a quantitative RT-PCR and calculating the fold change by the ΔΔCT method, a 20-fold or 3-fold upregulation of PLVAP transcripts was observed in iHCPEnC exposed to PMA or VEGF, respectively (Figure 4B and see Table S4). The stimulation with PMA leads to a significantly stronger expression of PLVAP compared to stimulation with VEGF (p = 0.03). We further focused on the PLVAP expression under untreated conditions or following exposure to either PMA or VEGF, as mentioned previously. Western blotting of a total protein lysate using a mouse monoclonal and a rabbit polyclonal antibody against PLVAP, showed that PLVAP is expressed in iHCPEnC, even in nontreated cells, and expression is upregulated in iHCPEnC after treatment with PMA or VEGF (Figures 4C and 4D). In immunofluorescence, PLVAP showed a punctate appearance with an increased punctate staining in PMA-treated and VEGF-treated cells (Figure 4E). The majority of the punctate PLVAP colocalized with caveolin-1 (Stan et al., 2004) and showed increased intensity to the periphery of the cell (Figure 4F).

**Figure 5:**
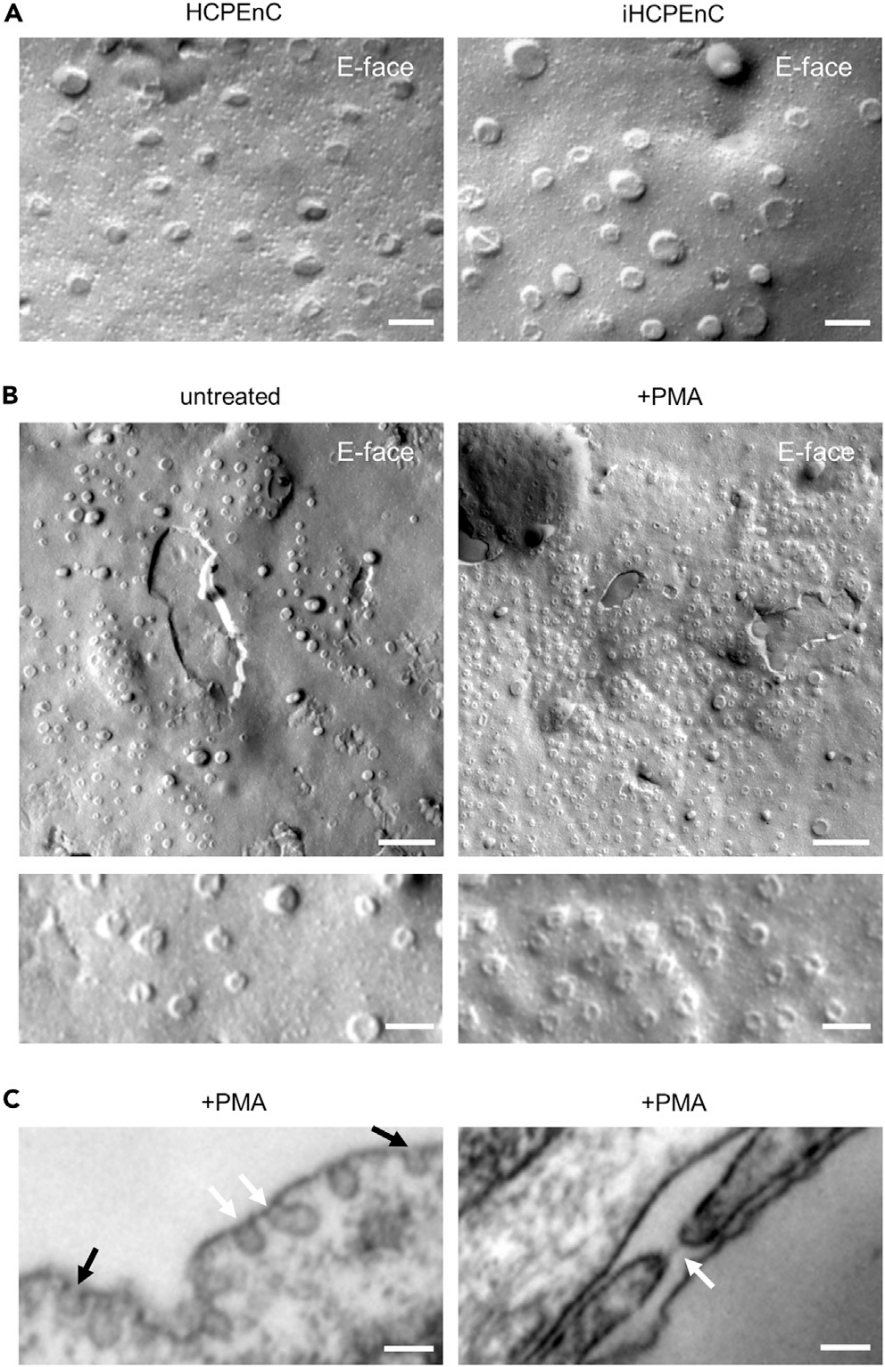
Comparative structural analysis of fenestrae in iHCPEnC (A) Comparative overview of freeze-fracture electron microscopy of primary and iHCPEnC PMA-treated cells. Scale bar, 100 nm. (B) Upper panels: Overviews of freeze-fracture electron microscopy of untreated and PMA-treated iHCPEnC. Clusters of PLVAP-associated structures (TEC, caveolae, and fenestrae) appear as circular elevation on the E-face of an iHCPEnC (upper panel). Scale bar, 500 nm. Lower panels: Details of freeze-fracture E-faces are shown. Scale bar, 100 nm. (C) Transmission electron microscopy of PMA-treated iHCPEnC. Caveolar structures showing stomatal diaphragms (white arrows, left panel) are compared to caveolar structures without diaphragms (black arrows, left panel), and a fenestral structure (right panel, arrow) was detected. Scale bar, 100 nm.

To further confirm the presence of PLVAP-associated structures as fenestrae, TEC, and/or caveolae, cells were analyzed by freeze-fracture electron microscopy and transmission electron microscopy (TEM). As shown in Figure 5A, structures appear as circular elevations predicting the presence of fenestrae, TEC, and/or caveolae in primary HCPEnC and iHCPEnC, with a similar morphology. PMA treatment showed a significant increase of these structures (Figure 5B). A further analysis by TEM revealed the existence of caveolar structures presenting stomatal diaphragms. In addition, a fenestral structure covered by a diaphragm was detected (Figure 5C).

### Establishment of a two-cell type *in vitro* model containing both endothelial and epithelial cells of the human CP

**Figure 6:**
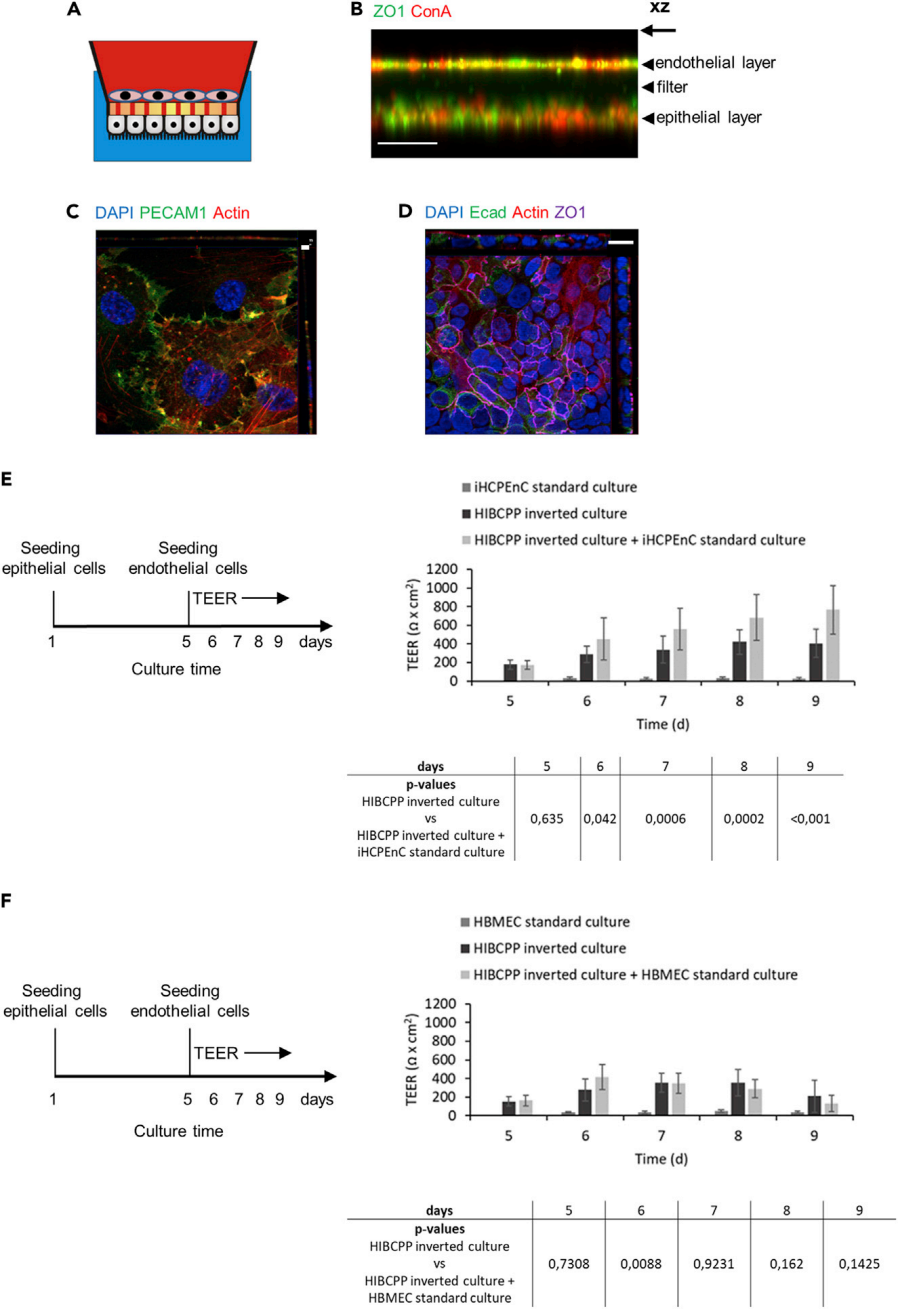
Schematic view, immunofluorescence analysis and functional analysis of the two-cell type model (A) Schematic representation of the *in vitro* two-cell type coculture model, composed of a blood compartment (red) containing the iHCPEnC, and the CSF side (blue) with the HIBCPP cells. (B) Immunofluorescence analysis of the two-cell type model using spinning disc confocal microscopy in XZ view shows the endothelial and epithelial cell layers stained with an antibody against ZO1 (green) and the plasma membrane marker Con A rhodamine (red). Scale bar, 30 μm. (C) Orthogonal view of the endothelial cell side of the coculture model. The endothelial cell layer was analyzed by immunofluorescence analysis using an anti-PECAM1 antibody (green), and actin was visualized with phalloidin Alexa 647 (red). Scale bar, 5μm. (D) Orthogonal view of the epithelial cell layer. HIBCPP cells were stained with an anti-Ecad antibody (green), an anti-ZO1 antibody (magenta), and actin was visualized with phalloidin Alexa 647 (red). Scale bar, 10 μm. (E) Experimental workflow and functional analysis of a two-cell type *in vitro* model composed of HIBCPP cells and iHCPEnC. HIBCPP cells were grown in the inverted culture, at day 5 iHCPEnC were seeded on the upper side of the filter membrane. The TEER developed by HIBCPP cells grown in coculture with iHCPEnC over 4 days (until day 9) was compared to the TEER developed by HIBCPP cells or iHCPEnC cultured alone. Below the graph, the p values for the statistical comparison of HIBCPP cells alone with HIBCPP cells in coculture are indicated. At least n = 3 biological with at least 3 technical replicates were included into the analysis. Data are represented as mean ± SEM. (F) Experimental workflow and functional analysis of a two-cell type *in vitro* model composed of HIBCPP cells and HBMEC. HIBCPP cells were grown in the inverted culture, at day 5 HBMEC were seeded on the upper side of the filter membrane. The TEER developed by HIBCPP cells grown in coculture with HBMEC over 4 days (until day 9) was compared to the TEER developed by HIBCPP cells or HBMEC cultured alone. Below the graph, the p values for the statistical comparison of HIBCPP cells alone with HIBCPP cells in coculture are indicated. At least n = 3 biological with at least 3 technical replicates were included into the analysis. Data are represented as mean ± SEM.

We have described a functional human *in vitro* model of the BCSFB based on HIBCPP cells grown on the bottom side of the filter in an inverted cell culture filter insert model (Schwerk et al., 2012; Dinner et al., 2016). We now extended this model by additionally cultivating iHCPEnC on the upper side of the filter, generating a two-cell type model as shown schematically in Figure 6A. At confluence of the iHCPEnC, the filter was subjected to indirect immunofluorescence using the marker ZO1 to detect cell contacts, and the membrane marker concanavalin A (Con A) rhodamine to visualize cellular boundaries (Figures 6B and S1). Immunofluorescence analysis of the two-cell type model in XZ view revealed an endothelial monolayer and the HIBCPP cell layer, respectively (Figures 6B and S1). The endothelial cell layer was confirmed by immunofluorescence against PECAM1 (Figure 6C), whereas for confirmation of the HIBCPP cells staining against Ecad was performed (Figure 6D).

### A two-cell type model based on iHCPEnC and HIBCPP cells displays consistently enhanced barrier function

The barrier integrity of the two-cell type *in vitro* model based on iHCPEnC and HIBCPP cells was assessed by TEER evaluation during cocultivation over 4 days. The TEER of the two-cell type model was significantly and consistently increased compared to the cultures containing only the epithelial HIBCPP cells or the endothelial iHCPEnC (Figure 6E). Importantly, this increase is higher than the additive TEER values of the endothelial and epithelial layers cultivated separately. A maximum of 700 Ω cm^2^ was reached at day 5 in the two-cell type model, whereas HIBCPP cells and ihCPEnC alone displayed a maximum of 400 Ω cm^2^ or below 50 Ω cm^2^, respectively. Importantly, when iHCPEnC were replaced by HBMEC, no consistent significant increase of the TEER was observed in the two-cell model compared to HIBCPP cells cultured alone (Figure 6F).

## Discussion

Here, we describe the generation and phenotypic characterization of an immortalized human CP endothelial cell line, which we termed iHCPEnC. To generate iHCPEnC, a lentiviral vector that transduces the catalytic subunit of hTERT was used—an approach suited to preserve the primary endothelial phenotype and to avoid oncogenic transformation—but still able to immortalize the cells (Bodnar et al., 1998). However, exogenous expression of hTERT can lead to activation of the c-Myc oncogene in some cells (Wang et al., 2000). Importantly, no elevated c-Myc expression in iHCPEnC was observed. iHCPEnC expressed the typical pan-endothelial markers PECAM1 and VWF, showed no phenotypic changes over 30 passages (60 population doublings), and maintained a normal endothelial phenotype, including the capacity to form capillary-like networks in Matrigel. In addition, a transcriptome comparison of iHCPEnC with primary HCPEnC revealed a high similarity.

It is assumed that endothelial cells undergo organ-specific differentiation that would imply differentiated gene expression profiles and changes in gene expression level. A comparison of the transcriptome of iHCPEnC with the primary HCPEnC revealed a high similarity, indicating that iHCPEnC maintained the expression profile of CP endothelial cells to a large extent. Interestingly, comparison of the transcriptome of iHCPEnC with that of HBMEC or HUVEC, endothelial cells derived either from the blood-brain barrier-derived or from veins from the umbilical cord, pointed to considerable differences.

GSEA comparing primary HCPEnC with iHCPEnC resulted in the identification of 805 gene sets, whereas GSEA comparing iHCPEnC with HBMEC or HUVEC identified 820 and 870 gene sets, respectively. Generation of heatmaps with genes obtained from selected gene sets further pointed to distinct gene expression profiles between the different endothelial cell types. We found genes involved in the “endothelial cell apoptotic process” were higher expressed in iHCPEnC than in HUVEC. Importantly, genes involved in “transport across blood-brain barrier” were expressed much stronger in HBMEC than in iHCPEnC. These observations support that iHCPEnC display a unique expression profile and are well suited as an endothelial component of CP tissue models, especially for, but not limited to, models intended for applications in pharmacological research.

A specific and functionally important structural feature of the CP endothelium is the fenestration that distinguishes it from other endothelia, such as that of the brain vasculature forming the BBB (Langen et al., 2019). Fenestrae are cellular openings of approximately 100 nm diameter that are sealed by diaphragms formed by assemblies of PLVAP (Schlingemann et al., 1985; Stan et al., 2004). In addition to fenestrae, PLVAP is also a component of caveolae and TEC (Bosma et al., 2018; Stan et al., 1997, 1999, 2004). It has been observed that primary fenestrae-containing endothelial cells lose these structures during immortalization as described for liver sinusoidal endothelial cells (Xie et al., 2013). In contrast, iHCPEnC expresses PLVAP even in the absence of any stimuli and maintains the presence of PLVAP-associated structures despite immortalization.

In mature brain microvascular endothelial cells forming a functional BBB, no expression of PLVAP was detected, but an increased expression of PLVAP was observed under pathological conditions, such as ischemia (Shue et al., 2008) or tumors of the brain (Carson-Walter et al., 2005). This indicates that absence of PLVAP is associated with barrier function at the BBB, whereas expression supports vascular leakiness. PLVAP is expressed in fenestrated cells, e.g., in the liver endothelium (Schlingemann et al., 1985; Auvinen et al., 2019; Terkelsen et al., 2020) or glomeruli of Wilms` tumor (Sarawar et al., 1988).

PMA is known to induce the formation of diaphragmed fenestrae, TEC, and caveolae in primary endothelial cells in culture (Lombardi et al., 1987; Stan et al., 2004). VEGF is also essential for formation and maintenance of diaphragmed fenestrae (Roberts and Palade, 1995). Moreover, PMA induces expression of PLVAP via VEGF and its receptors VEGFR1 and VEGFR2 in HUVEC (Strickland et al., 2005; Hamilton et al., 2019), whereas treatment with VEGF alone did not cause PLVAP expression in these cells (Hamilton et al., 2019). In contrast to previous observations in HUVEC, iHCPEnC revealed a basal PLVAP expression which increases when exposed to PMA or VEGF, suggesting cell line responsiveness to stimuli.

Altogether, our observations support iHCPEnC as a genuine model of the CP endothelium. The CP is considered as a multifunctional organ involved in processes as diverse as CSF production, barrier function, brain development, aging, and neuronal infections and diseases (Wolburg and Paulus, 2010; Kaur et al., 2016; Lun et al., 2015). We have described an *in vitro* model of the human BCSFB that faithfully recapitulates the barrier function of the CP (Schwerk et al., 2012). Still, an important role in maintaining the functions of organs, such as the CP, is played by the vasculature and requires the presence of endothelial cells with organ-specific characteristics (Augustin and Koh, 2017). Because iHCPEnC maintain the organ-specific characteristics of the CP endothelium, we combined iHCPEnC with HIBCPP cells to develop an advanced two-cell type *in vitro* model of the CP.

It is known that the CP epithelial cells play an important role during barrier function (Schwerk et al., 2015). In contrast, the fenestrated CP endothelium is not thought to contribute to the barrier, but to our knowledge, this assumption has not been tested with CP endothelial cells *in vitro*. We showed that iHCPEnC express certain tight junction associated proteins, such as ZO1, OCLN, CLDN1, and CLDN5. Although CLDN1 revealed a weaker immunofluorescence signal, the CLDN5 signal was increased. This observation is in agreement with *in situ* histology of CP endothelia of rats (Lippoldt et al., 2000). Although these results provide evidence of a tight junction organization by iHCPEnC, the cells did not develop a considerable TEER when grown on filter supports, which distinguishes them from brain microvascular endothelial cell lines such as HBMEC and hCHMEC/D3 (Weksler et al., 2005; Stins et al., 2001).

Interestingly, when HIBCPP cells were grown together with iHCPEnC in the two-cell *in vitro* model, consistently enhanced TEER values were reached that went beyond a simple additive effect of the two cell layers involved. Importantly this effect was specific for iHCPEnC, because a similarly enhanced TEER was not observed consistently when iHCPEnC were substituted with HBMEC. It has been shown that crosstalk between endothelial and epithelial cells in the lung can induce changes in barrier function, exemplified by a significantly increased TEER of 2260 ± 64 Ω cm^2^ compared to endothelial or epithelial cell monolayers alone (80 ± 12 Ω cm^2^ or 1400 ± 70, respectively (Chowdhury et al., 2010). The increase in TEER was correlated with the increase of OCLN shown by increased RNA and protein level (Chowdhury et al., 2010). Several tight junctions and adherens junction proteins are expressed in the BCSFB epithelial cells ensuring the barrier function. Tight junctions localize at the apical side, the adherens junction proteins JAM-A and Ecad were found at the basolateral compartment of the cell and display a functional relationship (Ando-Akatsuka et al., 1999). Both junctional protein complexes are highly dynamic structures which can assemble in response to stimuli inducing epithelial barrier formation.

Among these stimuli are cytokines derived from the endothelial cells (Verma et al., 2006). We have to consider several mechanisms of tight junction protein regulation in HIBCPP cells by iHCPEnC derived stimuli. These could impact at the gene expression level, at intracellular localization or on posttranslational modifications (phosphorylation, ubiquitination, and SUMOylation). To elucidate the exact mechanisms how iHCPEnC influence the barrier function of HIBCPP cells, further studies including RNA-seq as well proteomics analyses will be necessary. Our data indicate that the CP endothelium, although it does not contribute by developing a considerable TEER itself, contributes to the barrier function at the CP by affecting the CP epithelium.

### Limitations of the study

In this study, iHCPEnC have been characterized until 60 population doublings, which correspond to approximately 30 passages. For relevant *in vitro* research on functions in the context of the CP, it is essential that during the experimental setup the endothelium mirrors the properties of the primary HCPEnC. Therefore, iHCPEnC of a passage number below 30 should be used until preservation of CP endothelial features has been confirmed for later passages.

It is our goal to progressively integrate stroma and immune cells into our CP model, but currently studies requiring a complete CP structure might nevertheless require animal models, although differences between human and animal CP properties have to be considered. The CP plays important roles in the pathogenesis of CNS infections, neurodegenerative diseases, and brain metastasis of cancer cells. In this regard, the models presented by us constitute sophisticated tools for pharmacological applications covering transport of drugs across the BCSFB, a highly relevant aspect e.g., during the treatment of CNS tumors.

## STAR★Methods

### Key resources table

**Table:** 

REAGENT or RESOURCE	SOURCE	IDENTIFIER
**Antibodies**		

Rabbit anti-PLVAP	Abexxa	abx 129030
Mouse anti-PLVAP (clone 174/2)	Abcam	ab81719; RRID: AB_1658370
Mouse anti-PECAM1	Dako	M0823; RRID: AB_2114471
Rabbit anti-VWF	Abcam	ab6994; RRID: AB_305689
Rabbit anti-CLDN1	GeneTex	GTX134842; RRID: 2887357
Rabbit anti-CLDN5	Abcam	34-1600; RRID: AB_2533157
Rabbit anti-ZO1	Thermo Fisher	61-7300; RRID: AB_2533938
Rabbit anti-OCLN	GeneTex	GTX114949; RRID: AB_11177242
Rabbit anti-CAV1	Invitrogen	PA5-17447; RRID: AB_10985023
Rabbit anti-hTERT	Rockland	600-401-252; RRID: AB_2201591
Mouse anti-c Myc	NOVUSBIO	NB600-302SS; RRID: AB_ 2037063
Mouse anti-Ecad	BD Biosciences	610182; RRID: AB_397580
Mouse anti-β actin	Thermo Fisher	MA1-140; RRID: AB_2536844
Donkey anti-mouse Alexa 488	Thermo Fisher	A21202; RRID: AB_141607
Donkey anti-rabbit Alexa 488	Thermo Fisher	A21206; RRID: AB_2535792
Goat anti-mouse Alexa 594	Thermo Fisher	A11032; RRID: AB_2534091
Goat anti-mouse HRP	Thermo Fisher	62-6520; RRID: AB_2533947
Donkey anti-rabbit HRP	Merck	AP182P; RRID: AB_92591
Donkey anti-mouse Alexa 647	Thermo Fisher	A31571; RRID: AB_162542

**Biological samples**		

VEGFA	Novus Biologicals	NBP253040
Total RNA HBMEC	ScienCell	1005
Total RNA HUVEC	ScienCell	8005

**Chemicals, recombinant proteins**		

Agar-agar-Kobe I	Roth	5210.2
Collagenase, type I	Sigma	C130
Dispase ll	Sigma	D4693
ECM Matrigel	Sigma	E1270
Complete Classic Medium^TM^	Cell Systems	4Z0-500
Attachment Factor^TM^	Cell Systems	4Z0-201
Trypsin/EDTA 0.25%	Gibco	25200-056
Penicillin Streptomycin	Gibco	15070-063
GeneRuler (100bp)	Thermo Fisher	0243
Protein Standards	Thermo Fisher	P/N57318
Concanavalin A rhodamine	Vector Labs	RLK-2200
Dulbeccós PBS (1×)	Gibco	14190-094
Puromycin	Gibco	A11138-03
Polybrene	Santa Cruz	sc-134220
Triton X-100	Sigma	T8787
ProLong antifade reagent	Invitrogen	P34930
Phorbol-12-myristat-13-acetat	Sigma	P8139
Paraformaldehyde, 4% in PBS	Thermo Fisher	J19943
Trypan blue, 0.4%	Sigma	93595
RIPA Lysis buffer (10×)	Merck	20-188
Laemli Sample buffer	BioRad	161-0737
Milk powder	BioFroxx	1172GR500
Glutaraldehyde, 8%	Polysciences	00216-30
Bovine serum albumin, V	Merck	12659
Inulin FITC,	Sigma	F3272
DMEM/F-12	Gibco	11039-021
Phalloidin Alexa 568	Invitrogen	A12380
MOPS SDS running buffer	Novex	NP0001
Transfer buffer	Novex	NP0006-1
Tris/Glycine buffer (10×)	BioRad	161-0734
Protease inhibitor cocktail	Sigma	P2714
Critical commercial kits		
RNeasy mini kit	Qiagen	74104
Immobilon Western kit	Millipore	WBKLS0100
Brilliant IISYBR Green QPCR Mix	Agilent Technologies	600828
Lenti-Pac HIV Expression Packaging kit	Biocat	HPK-LvTR-20
Affinity Script QPCR cDNA synthesis kit	Agilent Technologies	600559
RNA Nano 6000 Assay Kit of the Bioanalyzer 2100 system	Agilent Technologies	5067-1513
Qubit® RNA Assay Kit	Life Technologies	Q33221
DNA High Sensitivity kit	Agilent Technologies	5067-4626
Qubit® DNA High Sensitivity kit	Life Technologies	Q32851

**Experimental model: cell lines**		

Human primary choroid plexus endothelial cells (HCPEnC)	This paper	
Human choroid plexus papilloma (HIBCPP)	Hiroshi Ishikawa	(Ishiwata et al., 2008)
Immortalized human choroid plexus endothelial cells (iHCPEnC)	This pape	
Human brain microvascular endothelial cells (HBMEC)	Kwang S Kim	John Hopkins, Maryland, USA

**Oligonucleotides**		

PLVAP FW (5′-3′) CCTTGAGCGTGAGTGTTTCCA RW (5′-3′) GGCAGGGCTGGGAGTTG	Sigma	(Wang et al., 2014)
ESM1 FW (5′-3′) GCCCTTCCTTGGTAGGTAGC RW (5′-3′) TGTTTCCTATGCCCCAGAAC	Sigma	(Jin et al., 2020)
MFSD2A FW (5′-3′) CTCCTGGCCATCATGCTCTC RW (5′-3′) GGCCACCAAGATGAGAAA	Sigma	(Vargas et al., 2014)
ACTA2 FW (5′-3′) CCAGAGCCATTGTCACACAC RW (5′-3′) CAGCCAAGCACTGTCAGG	Sigma	(Hu et al., 2019)
VEGFR1 FW (5′-3′) CAGGCCCAGTTTCTGCCATT RW (5′-3′) TTCCAGCTCAGCGTGGTCGTA	Sigma	(Zhao et al., 2007)
VEGFR2 FW (5′-3′) CCAGCAAAAGCAGGGAGTCTGT RW (5′-3′) CCAGCAAAAGCAGGGAGTCTGT	Sigma	(Zhao et al., 2007)
VEGFR3 FW (5′-3′) CCTGAAGAAGATCGCTGTTC RW (5′-3′) GAGAGCTGGTTCCTGGAGAT	Sigma	(Kumaravel et al., 2020)
LYVE1 FW (5′-3′) TGTTGCTTCTCACTTCCATCT RW (5′-3′) GCTTGGACTCTTGGACTCTTC	Sigma	(Gao et al., 2006)
GAPDH FW (5′-3′) AGTCAGCCGCATCTTCTT RW (5′-3′) GCCCAATACGACCAAATCC	Sigma	(Severino et al., 2017)
ZO1 FW (5′-3′) GCCAAGCAATGGCAGTCTC RW (5′-3′) CTGGGCCGAAGAAATCCCATC	Sigma	(Schwerk et al., 2012)
Occludin FW (5′-3′) AGGAACACATTTATGATGAGCAG RW (5′-3′) GAAGTCATCCACAGGCGAA	Sigma	(Schwerk et al., 2012)
Claudin1 FW (5′-3′) GAAGATGAGGATGGCTGTCA RW (5′-3′) AAATTCGTACCTGGCATTGA	Sigma	(Schwerk et al., 2012)
E-Cadherin FW (5′-3′) CCTGCCAATCCCGATGA RW (5′-3′) TGCCCCATTCGTTCAAGTA	Sigma	(Schwerk et al., 2012)
Claudin5 FW (5′-3′) AGGCGTGCTCTACCTGTTTTG RW (5′-3′) AACTCGCGGACGACAATGTT	Sigma	(Ichikawa-Shindo et al., 2008)

**Software and algorithms**		

Image*J*/FIJI	NIH, Bethesda, Maryland, USA	http://ImageJ.nih.gov/ij/
Imaris 9.6.0	EMBL	http://imaris.oxinst.com/
Chemi-Capt5000	Vilber Lourmat	www.vilber.com
I-control 2	TECAN	https://lifesciences.tecan.com/software
Zen	Carl Zeiss	https://www.zeiss.de/mikroskopie/produkte/mikroskopsoftware/zen.html
NGS analysis plackage systemPipeR	(Backman and Girke, 2016)	https://bioconductor.org/packages/systemPipeR/
FastQC	Babraham Bioinformatics	https://www.bioinformatics.babraham.ac.uk/projects/fastqc/
trim_galore software version 0.6.4	(Bray et al., 2016)	https://www.bioinformatics.babraham.ac.uk/projects/trim_galore/
limma package	(Ritchie et al., 2015)	https://bioconductor.org/packages/limma/
clusterProfiler	(Yu et al., 2012)	https://bioconductor.org/packages/clusterProfiler/
complexHeatmap version 2.0.0	(Gu et al., 2016)	https://bioconductor.org/packages/ComplexHeatmap/
kallisto version 0.46.1	Bray et al., 2016	https://github.com/pachterlab/kallisto/issues/240
SAS, release 9.4	SAS institute Inc., Cary, North Carolina	https://support.sas.com/software/94/

This study generate RNA sequencing datasets available on the following link: https://www.ncbi.nlm.nih.gov/geo/query/acc.cgi?acc=GSE198846. The raw and normalized data are deposited in the Gene Expression Omnibus database (http://www.ncbi.nlm.nih.gov/geo/; accession No. GSE198846).

### Resource availability

#### Lead contact

Further information and requests for generated resources or reagents should be directed and will be fulfilled by the Lead Contact, Dr. Walter Muranyi (walter.muranyi@medma.uni-heidelberg.de).

#### Materials availability

The cell line generated newly in this study, iHCPEnC, is available upon request and following standard Material Transfer Agreement (MTA), due to our institutional recommendations.

### Experimental model and subject details

#### Cell lines and culture conditions

iHCPEnC were grown at 37 °C, 5% CO_2_ in Complete Classic Medium^TM^ for endothelial cells without phenol red (Cell Systems, Kirkland, USA) and supplemented with 10% fetal bovine serum, and CultureBoost^TM^ (Cell Systems, Kirkland, USA) containing growth factors, but without hydrocortisone, ascorbate and VEGF, and 1% penicillin/streptomycin (100 U/mL penicillin; 100 μg/mL streptomycin). Cells were grown continuously under 0.5 μg/mL puromycin und passaged routinely weekly 1:4.

HIBCPP cells were grown at 37°C, 5% CO_2_, in Dulbeccós modified Eagle’s medium (DMEM/F-12) supplemented with 10% FCS and penicillin/streptomycin (100 U/mL penicillin; 100 μg/mL streptomycin).

#### Human tissues

The human CP sample used for isolation of the primary HCPEnC was isolated from an 18 years old Japanese male and approved by the ethics committee of the University of Tsukuba, Japan (Approval-Nr. R1-058).

### Method details

#### Preparation of endothelial cell from human choroid plexus

The choroid plexus (CP) tissue from a lateral ventricle was washed with Hanks’ solution and digested in a buffer containing 0.1% trypsin and 0.02% EDTA/PBS (−) for 15 min at 37°C. Tissue pieces were washed again in Hanks’ solution, centrifuged at 430 ×g for 5 min and then incubated with 0.25% crude collagenase (Collagenase C0130, type 1, Funakoshi, Japan) and 0.2% Dispase (DispaseII, Funakoshi, Japan)/Hanks’ solution at 37°C for 30 min. The sediment was resuspended in endothelial growth medium (Complete Classic Medium, Cell Systems, Kirkland, USA) and then seeded onto Attachment Factor^TM^ (Cell Systems, Kirkland, USA) coated dishes. Cells obtained from the incubation, were cultivated on Matrigel (50 μg/cm^2^) coated dishes, at a density of 1 × 10^5^ cells/mL in presence of 20 ng/mL vascular endothelial growth factor (VEGF) for 48 h. The capillary-like tubes were then isolated using microforceps under a stereoscopic microscope, dissociated with 0.1% trypsin–0.02% EDTA/PBS (−) and after centrifugation (430 × g for 5 min), cells were cultivated in Complete Classic Medium for endothelial cells without phenol red (Cell Systems, Kirkland, USA). When fibroblasts or pavement-like cell colonies were observed, cultivation of cells with VEGF at 30 ng/mL was performed.

#### Immortalization, cell transformation and *in vitro* angiogenesis assay

The immortalization of primary human CP endothelial cells was performed using lentiviral particles transducing hTERT (Biocat, Germany) according to manufacturer’s protocol. Prior to transduction experiments, the puromycin concentration that led to 100% of endothelial cell death was determined. Cells were seeded at a density of 2 × 10^4^ per 24 well plate and 24 h later subjected to viral infection at a concentration of 1 × 10^4^ TU (transduction units)/well in the presence of Polybrene (Santa Cruz Biotechnology) at a final concentration of 8 μg/mL. After 2 h, cells were washed and incubated for 4 days in endothelial classic medium. Cells were trypsinized and re-seeded on 6-well plates and incubated for additional 48 h and subjected to 1.0 μg/mL puromycin (Gibco) for selection. Puromycin-resistant colonies usually emerged from single cells and were further subjected to analysis of endothelial morphology and expression of endothelial markers and contact inhibition at confluent cell layer. The cell line was grown on Attachment Factor^TM^-coated plates in endothelial complete classic medium in the presence of 0.5 μg/mL puromycin and the splitting ratio was 1:4.

To determine the population doubling time, 4 × 10^4^ cells were seeded on a coated 6-well plate and cultivated in endothelial classic medium. Cells were counted daily for 6 days using a hemocytometer in the presence of trypan blue (Sigma). From the linear part of the growth curve, cell number versus time and the population doubling time (PDT) was calculated with the equation:

PDT=(t2−t1)/3.32×(logn2−logn1)

where t is time and n is number of counted cells.

To determine whether iHCPEnC are capable to grow anchorage-independent in soft agar, three dilutions of 1,000, 2,000 and 4,000 cells/mL were resuspended in Complete Classic Medium with 0.4% agar and poured in a 24-well tissue plate on a layer of 0.6% solidified base agar in the same medium (Cox and Der, 1994). The cells were counted with a hemocytometer in the presence of trypan blue staining. The plates were incubated in a humidified 5% CO_2_ incubator at 37°C for up to 4 weeks. The human glioblastoma U87 and HeLa cells were used as positive control (Murphy et al., 1992). Colony forming was examined weekly.

Matrigel (Sigma) was layered into 48-well tissue plate and coated with 100 μL/well and allowed to gel at 37°C. iHCPEnC were resuspended in fresh medium at 500, 1000 and 2000 cells/mL, and 1 mL aliquots were layered over a preformed gelled layer of extracellular Matrigel in wells of a 48-well culture plate and incubated at 37°C for 48 h. Development of capillary-like networks and tubular structures were imaged by phase-contrast microscopy.

#### Immunofluorescence and imaging

Primary and immortalized CP endothelial cells were seeded on Attachment Factor^TM^ coated glass coverslips or coated 8-chambered Lab-Tek (Nunc) slides and grown to confluence. For characterization of endothelial marker and tight junction proteins, cells were washed and fixed 20 min with 4% paraformaldehyde (PFA) in PBS (Thermo Fisher, Germany), washed and blocked for 10 min with 2% bovine serum albumin (BSA) in PBS. Subsequently, cells were permeabilized with 0.1% Triton X-100/PBS for 5 min, washed and blocked with 2% BSA/PBS. The cover slips were incubated with appropriate primary antibodies diluted in 2% BSA/PBS. The following dilutions were used: mouse monoclonal anti-PLVAP (clone 174/2) (1:200), mouse anti-PECAM1 (1:200), rabbit anti-VWF (1:300), rabbit anti-CLDN1 (1:200), rabbit anti-CLDN5 (1:200), rabbit anti-ZO1 (1:400), rabbit anti-OCLN (1:200), rabbit anti-CAV1 (1:200). Detection of hTERT was performed using a rabbit anti-hTERT (1:200) antibody. Alternatively, cells were fixed and permeabilized with ice-cold methanol, washed in PBS, and blocked in 2% BSA/PBS before incubating with the first antibody. The washing and blocking procedures were repeated before incubation with appropriate secondary antibodies diluted in 2% BSA/PBS. DNA was visualized with 4,6-diamidino-2-phenylindole dihydrochloride (DAPI; Sigma). To confirm the specificity of the fluorescent signal, immunofluorescence without primary antibody were performed. Coverslips were mounted in ProLong gold antifade reagent (Invitrogen). PMA (Merck, German) and VEGF (VEGFA, Novus Biologicals) treatment of cells was performed for 3 day and commonly (Hamilton et al., 2019) used concentrations of 50 nM and 40 ng/mL, respectively.

#### Isolation of RNA and quantitative RT-PCR

Total RNA was extracted from primary and immortalized human CP endothelial cells using RNeasy Mini kit (Qiagen, Germany) according to the manufacturer’s instructions. For reverse transcription an AffinityScript QPCR cDNA Synthesis kit (Agilent Technologies, Germany) was used. The reverse transcription was performed in a reaction volume of 20 μL containing 1 μg of total RNA and oligo dT primer. Primer annealing was performed at 25°C for 5 min and reverse transcription was run at 42°C for 1 h, followed by inactivation of the enzyme at 95°C for 5 min. One microliter of cDNA reaction product was used for PCR amplification using the Brilliant II SYBR Green QPCR Mastermix (Agilent Technologies, Germany). Amplification was performed in a Thermo cycler MX3005P™ (Agilent Technologies, Germany) with an initial denaturation step at 94°C for 15 min; 40 cycles of 94°C for 30 s, 60°C for 30 s and 72°C for 30 s, and a final elongation step at 72°C of 5 min. The sequence of the primers used is listed in Table oligonucleotides. The fold change was calculated via the 2-ΔΔCT method using GAPDH as an internal control.

#### Western blot analysis

Protein expression was evaluated by immunoblot analysis. Cells were lysed in ice-cold 1× RIPA buffer (Merck) 50 mM Tris-HCl, pH 7.4, 150 mM NaCl, 0.25% deoxycholic acid, 1% NP-40, 1mM EDTA supplemented with sodium orthovanadate (1mM) and sodium fluoride (50 mM) and protease inhibitor cocktail (Sigma). Protein concentration of lysates was determined using BioRad assay. Protein lysates were resolved on a 4–12% acrylamide Bis-Tris gel (NuPAGE, Invitrogen) and transferred onto nitrocellulose membranes (BioRad). Membranes were blocked for 1 h in 5% milk powder solution in PBS, and incubated with primary antibodies overnight at 4 °C, washed and incubated with an appropriate HRP-conjugated secondary antibody for 1 h at room temperature. The membranes were developed with Immobilon Western kit (Millipore). The following antibodies were used: mouse monoclonal against PLVAP (clone 174/2) (Abcam), rabbit polyclonal against PLVAP (Abbexa), mouse monoclonal anti c-Myc (clone 9E10) and mouse monoclonal against β-actin (Thermo Fisher).

#### Cell barrier integrity analysis

For barrier integrity studies, filter inserts with pore size of 0.4 μm at a pore density of 2 × 10^6^ cm^−2^ (culture area 33,6 mm^2^) (Millipore, Germany) coated with Attachment Factor^TM^ were used. Confluent cell layer was verified by phase contrast microscopy. TEER values of empty filter inserts were deducted. In addition, barrier integrity within time-course experiments was monitored through measurement of TEER using a tissue voltohmmeter (Millipore, Germany) and permeability analysis with FITC-Inulin (Sigma, Germany) as a tracer solution at a concentration of 100 μg/mL (Gründler et al., 2013). The FITC-Inulin (average molecular size of 3,000–6,000) was added to the upper filter compartment and the flow through to the lower compartment was determined using a Tecan Infinite M200 Multiwell reader (Tecan, Switzerland). Values are represented as mean ± SEM of triplicate filter.

#### Freeze fracture and transmission electron microscopy

For freeze-fracture EM analysis, filters were processed as described by (Greene et al., 2019). Cells fixed with 2.5% glutaraldehyde in 0.1 M cacodylate buffer overnight at 4 °C. After washing with cacodylate buffer, cells were cryoprotected in 30% glycerol and frozen in liquid nitrogen. After fracturing and shadowing with platinum and carbon (BAF400D; Balzers, Liechtenstein), organic material was removed by a sodium hypochlorite wash. The analysis was performed in a transmission electron microscope, Zeiss EM-10 (Oberkochen, Germany) and imaged with a digital camera (Tröndle GmbH).

For transmission electron microscopic analyses iHCPEnC cells were grown in Attachment Factor™ coated insert filters. Thin section electron microscopy was performed according to protocol (Brunner et al., 2020). In brief, filters were fixed over night at 4 °C, 2.5% glutaraldehyde solution in 100 mM cacodylate buffer (pH 7.4). Filters were washed twice with 100 mM cacodylate buffer (pH 7.4) fixed in 1% osmium tetroxide (OsO_4_) in cacodylate buffer for 1 h and dehydrated in ascending series of ethanol and propylene oxide. Contrast enhancement was done by bloc-staining in uranyl-acetate in 70% ethanol for 4 h and flat-embedding in Araldite (Serva, Heidelberg, Germany). Semi-(1 μm) and ultrathin sections (50 nm) were cut using an ultramicrotome (Ultracut R, Leica, Bensheim, Germany). Ultrathin sections were stained with lead citrate followed by mounting on copper grids and final analysis with a Zeiss EM-10 (Oberkochen, Germany) electron microscope.

#### Two-cell type *in vitro* model of the human CP

In order to establish a two-cell type co-culture system, composed of both an epithelial and an endothelial cell layer, filter inserts with pore size of 0.4 μm at a pore density of 2 × 10^6^ cm^−2^ (culture area 33,6 mm^2^) (Greiner Bio-One, #662641, Germany) were used. HIBCPP cells were cultured in DMEM/HAM’s F12 1:1 supplemented with 5 mg/mL insulin and 10% FBS. For assembly of an inverted HIBCPP cell culture the cells were seeded on the lower part of the membrane of a filter insert (Schwerk et al., 2015; Dinner et al., 2016). The inserts are flipped over into a 12-well-plate and filled from below with medium. After pre-wetting the upper part of the membrane, 8 × 10^4^ HIBCPP cells were seeded. 24h after seeding, inserts were flipped back into the right orientation into a 24 well plate. Usually, 5 days after seeding of the HIBCPP cells, at a TEER value of 100–300 Ω cm^2^ 4 × 10^4^ iHCPEnC cells were added to the upper compartment of the insert. 24h after seeding the iHCPEnCs, the TEER was monitored daily using a voltohmmeter equipped with an STX-2 electrode (Millipore, Schwalbach, Germany) (Schwerk et al., 2012).

When confluence was achieved, with TEER values ∼300 Ω cm^2^, the filters were fixed in 4% paraformaldehyde, washed, excised and subjected to indirect immunofluorescence. Following markers were used: mouse monoclonal against PECAM1 (DAKO), rabbit polyclonal against ZO1 (Invitrogen), mouse anti-Ecad (BD Biosciences), and phalloidin Alexa 568 (Invitrogen). Cell membranes were stained with Con A rhodamine (Vector Laboratories, USA). To image the whole two-cell type system, z-stacks from the top through the filter were imaged using an Olympus SpinSR10 spinning disk confocal (Olympus, Tokyo, Japan) equipped with an UPLXAPO 20×/0.8 NA air objective (Olympus), a CSU-W1 SoRa-Unit with a 50μm pinhole disk (Yokogawa, Tokyo, Japan) and an ORCA-Flash 4.0 V3 Digital CMOS Camera (Hamamatsu, Hamamatsu City, Japan). Images were processed using FIJI/ImageJ and Imaris 9.6.0. In addition, images were also taken with a Zeiss Axio Apotome and Zen software (Carl Zeiss, Germany) using a Plan-APOCHROMAT 63×/1,4 oil objective.

#### RNA sequencing

RNA integrity was checked using the RNA Nano 6000 Assay Kit of the Bioanalyzer 2100 system (Agilent Technologies, Santa Clara, CA), and concentration was measured with Qubit® RNA Assay Kit in Qubit® 2.0 Fluorometer (Life Technologies, Carlsbad, CA).

RNA sequencing was performed according to the manufacturer’s protocol (GenXPro GmbH, Germany). For mRNA sequencing analysis, 3 independent samples of each cell line prepared from total RNA was used. The cDNA synthesis was performed by reverse transcription starting from random hexamer oligonucleotides, followed by second strand synthesis and barcoding for Illumina sequencing, 2 × 75bp PE of 20 Mio Reads/library.

### Quantification and statistical analysis

For quantitative variables, mean values together with standard deviations, minima and maxima have been assessed, separated for each cell line and each day. Repeated measures analysis has been performed in order to investigate the impact of both factors (cell line and day) on the target variable and to test if the response profiles over time are different for the cell lines. In the case of significant interaction, 2 sample t tests have been performed to compare the 2 cell lines on each day. In general, a test result with a p value less than 0.05 has been considered as statistically significant. All these statistical calculations have been performed using SAS, release 9.4 (SAS institute Inc., Cary, North Carolina), USA).

For comparison of iHCPEnC stimulated with VEGF or PMA, respectively, a paired two-sided t-test was performed. A test result with a p value less than 0.05 has been considered as statistically significant.

Most of the bioinformatic procedure was done with R and bioconductor using the NGS analysis plackage systemPipeR (Backman and Girke, 2016). Quality control of raw sequencing reads was performed using FastQC (Babraham Bioinformatics). Low-quality reads were removed using trim_galore (version 0.6.4). The resulting reads were aligned to human genome version GRCh38.p13 from GeneCode and counted using kallisto version 0.46.1 (Bray et al., 2016). The count data was transformed to log2-counts per million (logCPM) using the voom-function from the limma package (Ritchie et al., 2015).

Differences of the log2-CPM of the samples were calculated and these differences were sorted and used as a ranked list for the GSEA.

The Gene Ontology analysis was made with clusterProfiler (Yu et al., 2012) package in R. Heatmaps were created using the complexHeatmap version 2.0.0 (Gu et al., 2016).

Principle Component Analysis (PCA) was performed with prcomp (stat package) in R. The plot was created with ggplot2 package.

## Data Availability

• This study did not generate computer algorithm or code. • This study generate RNA sequencing datasets available on the following link: https://www.ncbi.nlm.nih.gov/geo/query/acc.cgi?acc=GSE198846. The raw and normalized data are deposited in the Gene Expression Omnibus database (http://www.ncbi.nlm.nih.gov/geo/; accession No. GSE198846). • The GSEA comparing primary HCPEnC with iHCPEnC (Table S1, GSEA based gene sets identified between primary HCPEnC and iHCPEnC, Related to Figure 2C) • The GSEA comparing iHCPEnC with HBMEC (Table S2, GSEA based gene sets identified between iHCPEnC and HBMEC, Related to Figure 2B). • The GSEA comparing iHCPEnC with HUVEC (Table S3, GSEA based gene sets identified between iHCPEnC and HUVEC, Related to Figure 2B). Any additional information required to reanalyze the data reported in this paper is available from the lead contact upon request.

## Supplemental information

Document S1. Figures S1 and S4 and Table S4

Table S1. GSEA-based gene sets identified between primary HCPEnC and iHCPEnC, related to STAR Methods and Figure 2C

Table S2. GSEA-based gene sets identified between iHCPEnC and HBMEC, related to STAR Methods and Figure 2B

Table S3. GSEA-based gene sets identified between iHCPEnC and HUVEC, related to STAR Methods and Figure 2B
