# Supplementary material for: Immortalized human choroid plexus endothelial cells enable an advanced endothelial-epithelial two-cell type in vitro model of the choroid plexus
Source: iScience. 2022 May 10;25(6):104383. doi: 10.1016/j.isci.2022.104383 (PMC9133638; doi:10.1016/j.isci.2022.104383)
Supplement: Document S1. Figures S1 and S4 and Table S4 [file mmc1.pdf]

iScience, Volume *25*

Supplemental information

Immortalized human choroid plexus endothelial cells enable an advanced endothelial-epithelial two-cell type *in vitro* model of the choroid plexus

Walter Muranyi, Christian Schwerk, Rosanna Herold, Carolin Stump-Guthier, Marko Lampe, Petra Fallier-Becker, Christel Weiß, Carsten Sticht, Hiroshi Ishikawa, and Horst Schroten

SUPPLEMENTAL INFORMATION

Table S4, Quantification of representative iHCPEnC transcripts detected by RT-PCR, related to Figure 4

Figure S1, Immunofluorescence analysis of the two-cell type model using spinning disc confocal microscopy, related to Figure 6

Table S4. Quantification of representative iHCPEnC transcripts detected by RT-PCR

	PLVAP	ESM1	MFSD2A	ACTA2	VEGFR1	VEGFR2	VEGFR3	LYVE1	GAPDH
Fold change PMA	14,7	1,4	0,5	0,3	4,8	0,6	0,3	0,2	1
SD	3,63	0,47	0,16	0,20	1,41	0,13	0,07	0,08	0,00
Fold change VEGF	1,6	1,2	0,7	0,9	1,2	0,9	0,9	0,5	1,0
SD	0,38	0,07	0,05	0,27	0,08	0,10	0,10	0,03	0,00

iHCPEnC treated with PMA and VEGF for 3 days; SD, standard deviation.

Quantification of representative iHCPEnC transcripts detected by RT-PCR. iHCPEnC were seeded on a 6 well plate and grown to confluence. Cells were treated with PMA or VEGF for 3 days. Total RNA was extracted and subjected to RT-PCR. Two independent reactions were performed. Fold changes of transcripts after cell treatment with PMA and VEGF are shown. For PMA and VEGF treated cells n=2 and n=3, respectively, were used.

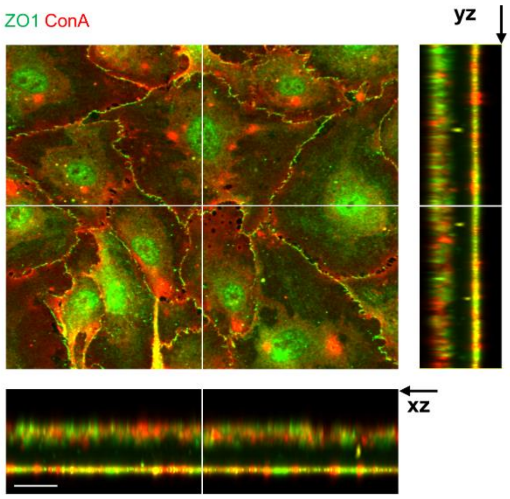

Figure S1. Immunofluorescence analysis of the two-cell type model using spinning disc confocal microscopy. The two-cell type model was stained with the tight junction marker ZO1 (green) and the membrane marker Con A rhodamine (red). Orthogonal view of the endothelial side of the two-cell type model, displaying the xy, yz and xz view of the model. Scale bar, 30 µm.
